# Hereditary Pheochromocytoma With a Mutation in the Succinate Dehydrogenase Subunit A Gene

**DOI:** 10.7759/cureus.24584

**Published:** 2022-04-29

**Authors:** Gowri Karuppasamy, Amer A Farooqi, Sadia Sajid, Elhadi Elouzi

**Affiliations:** 1 Internal Medicine, Hamad Medical Corporation, Doha, QAT; 2 Radiology, Hamad Medical Corporation, Doha, QAT

**Keywords:** genetics, succinate dehydrogenase, paraganglioma, cancer, adrenal, mutation, sdha, hereditary, pheochromocytoma

## Abstract

Pheochromocytomas and paragangliomas (PPGLs) are rare neuroendocrine tumors with diverse clinical presentations. Pathogenic variants in the genes encoding different subunits of the succinate dehydrogenase enzyme complex that plays a central role in energy metabolism have been linked to hereditary PPGL syndromes. Here we report a rare case of hereditary pheochromocytoma with a novel mutation in the succinate dehydrogenase subunit A (*SDHA*) gene.

A middle-aged woman presented with left-sided abdominal pain and was incidentally found to have bilateral adrenal lesions on abdominal imaging. Imaging characteristics were suggestive of pheochromocytoma. She denied any symptoms of catecholamine excess but her plasma metanephrines level was elevated. Iodine-131 metaiodobenzylguanidine (131I-MIBG) whole-body scan showed abnormal focal radiotracer uptake at the left adrenal gland, and she then underwent left-sided adrenalectomy. Following surgery, the patient had symptomatic relief and histopathology confirmed the diagnosis of pheochromocytoma. Genetic testing revealed that she was positive for a pathogenic mutation in the SDHA gene consistent with the diagnosis of hereditary PPGL syndrome.

The detection of susceptibility genes for hereditary PPGL syndromes has key implications, for surveillance to detect extra-adrenal disease and recurrent tumors, as well as for consideration of genetic testing for family members.

## Introduction

Pheochromocytomas and paragangliomas (PPGLs) are neuroendocrine tumors that arise from chromaffin cells in the adrenal gland or in extra-adrenal sympathetic and parasympathetic nerve ganglia. Recent advances in genetic testing have contributed to the diagnosis of hereditary PPGL syndromes. Alterations in genes that encode proteins in the mitochondrial complex II, particularly the succinate dehydrogenase enzyme complex that plays a central role in energy metabolism, have been identified as causative for PPGL syndromes. Here, we report a rare case of hereditary pheochromocytoma with a mutation in the succinate dehydrogenase subunit A *(SDHA)* gene.

## Case presentation

A 63-year-old woman, a chronic smoker with no past medical or surgical history, presented to the emergency department with worsening left upper abdominal pain for a few months. She denied any history of headache, sweating or palpitations. She did not take any regular medications. Family history was significant for breast cancer. Her mother was diagnosed with bilateral breast cancer at the age of 42 years and her sister was diagnosed with breast cancer at the age of 38 years. She had no family history of endocrine tumors.

**Figure 1:**
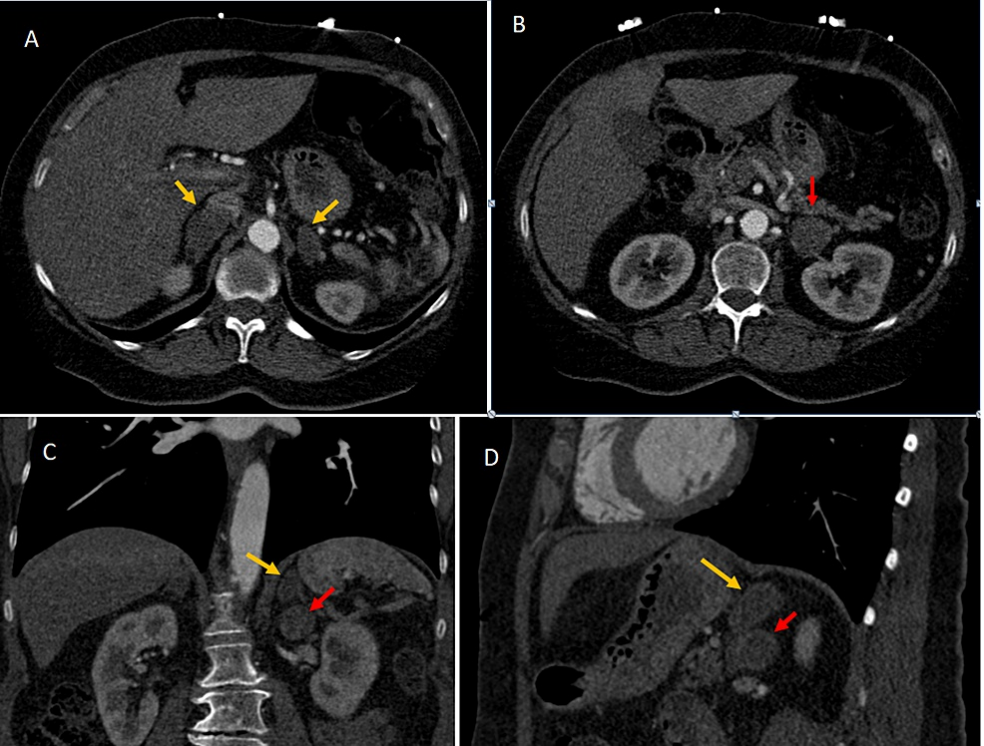
Post-contrast computed tomography (CT) scan abdomen (A) and (B) Axial, (C) Coronal, (D) Sagittal images: Bilateral bulky adrenal glands (orange arrows) with a 2.7 x 3.1 cm rounded lesion in the left renal hilar region (red arrow).

Physical examination was normal with a blood pressure of 123/78 mmHg. Routine laboratory investigations were unremarkable. Computed tomography (CT) scan of the abdomen showed bilateral bulky adrenal glands with a lesion in the left adrenal gland (Figure 1). 

**Figure 2:**
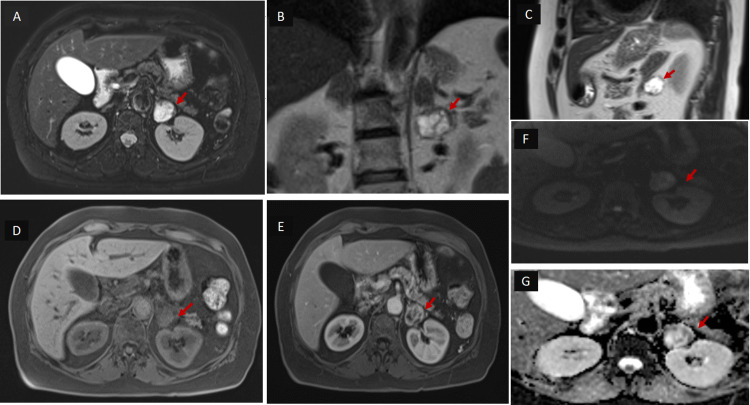
MRI abdomen: exophytic lesion (red arrow) measuring 2.8 x 2.6 cm inferior to the left adrenal gland abutting along the medial cortex of the left kidney with persistent fat planes (A), (B) and (C): Axial fat-saturation, Coronal and Sagittal T2WI: The lesion is hyperintense. (D) Axial T1WI: The lesion is iso to hypointense on T1.  (E) Axial post-contrast: heterogeneous enhancement. (F) and (G): DWI & ADC images: Subtle areas of diffusion restriction. T1W1: T1-weighted image, T2WI: T2-weighted image, DWI: Diffusion-weighted imaging, ADC: apparent diffusion coefficient image

**Figure 3:**
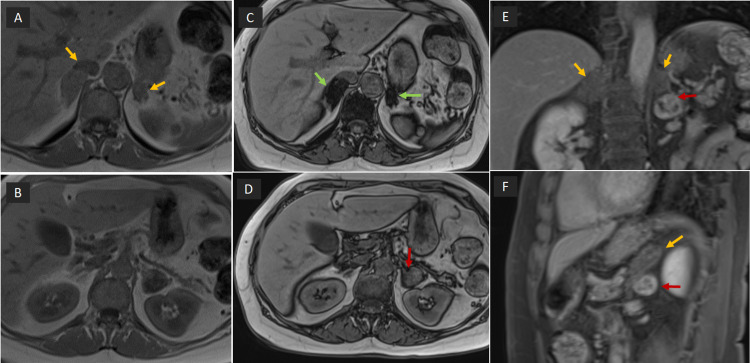
MRI abdomen (A) and (B) in phase, (C) and (D) out of phase images: Both adrenal glands are bulky (orange arrow), measuring 2.9 x 2 cm on the right and 2.5 x 1.5 cm on the left, with significant signal drop-off (green arrow) suggestive of adenomatoid hyperplasia. Exophytic left adrenal gland lesion does not show signal drop out (red arrow).  (E) and (F): Post-contrast Coronal and Sagittal images - no significant enhancement of bulky adrenal glands (orange arrow), heterogeneous enhancement of exophytic left adrenal gland lesion (red arrow).

Magnetic resonance imaging (MRI) abdomen was performed for further characterization - the lesion measured approximately 2.8 x 2.6 cm with imaging characteristics suggestive of pheochromocytoma (Figures 2, 3).

Additional laboratory investigations revealed that the plasma normetanephrines level was normal, but the plasma metanephrines level was elevated at 1.5 nmol/L (reference range <0.90 nmol/L). The 24-hour urine fractionated metanephrines level was normal. The dehydroepiandrosterone sulfate (DHEA-S) level was normal. An overnight 1 mg Dexamethasone Suppression Test (OnDST) was performed, which was normal. 

**Figure 4:**
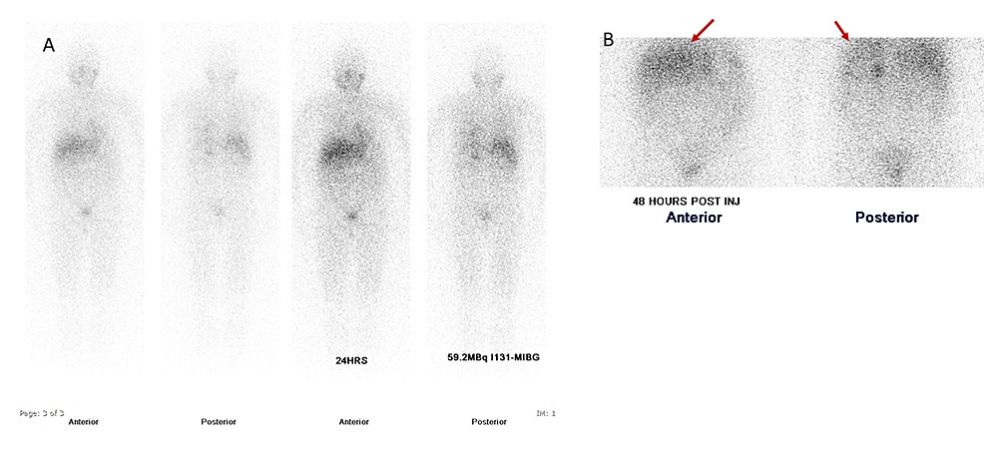
Iodine-131 metaiodobenzylguanidine (MIBG) whole-body anterior and posterior scan Abnormal focal radiotracer uptake at the left adrenal gland, persistent on the 24- and 48-hour scans (red arrow) signifying a neuroectodermal medullary adrenal tumor. No extra-adrenal pathological radiotracer concentration.

An Iodine-131 metaiodobenzylguanidine (131I-MIBG) whole-body scan showed abnormal focal radiotracer uptake at the left adrenal gland, persistent on the 24- and 48-hour scans, thus signifying a focal neuroectodermal medullary adrenal tumor (Figure 4). No extra-adrenal pathological radiotracer concentration was seen.

The patient underwent robotic excision of the left renal hilar mass. Subsequently, histopathology confirmed the diagnosis of pheochromocytoma measuring 2 x 2 x 1.5 cm with PASS (Pheochromocytoma of the Adrenal gland Scaled Score) score of 1/10 due to cellular monotony. The tumor cells were positive for vimentin, synaptophysin, chromogranin, CD56, Gata-3 and patchy BCL-2 but negative for Ae1/Ae3, Melan-A, calretinin, PAX8 and Inhibin. The sustentacular cells were positive for S100 and the Ki-67 proliferative index was low.

Following surgery, the patient's blood pressure remained normal. The 24-hour urine fractionated metanephrines level was repeated at 1 year and 2 years after surgery and remained normal. Plasma metanephrines could not be repeated as the test was not available in our hospital at the time. A follow-up CT scan of the abdomen revealed a 28-mm lesion of the right adrenal gland with no change compared to the previous CT. A residual lesion anterior to the upper part of the left kidney 18 mm in diameter was also noted. Repeat imaging after 2 years showed no interval changes in the bilateral adrenal lesions.

A screening mammogram showed focal asymmetry in the right breast with multiple tiny cysts in the right breast and a biopsy confirmed benign breast tissue. She was referred to a genetic counselor for DNA typing, which was negative for breast cancer genes (BRCA1 and BRCA2) but positive for a pathogenic mutation in the SDHA gene consistent with the diagnosis of hereditary paraganglioma syndrome.

She was counseled regarding the disease and the associated tumors and kept on routine surveillance including annual monitoring of 24-hour urine metanephrines level and endoscopic evaluation for gastrointestinal stromal tumors. Testing of first-degree relatives was also recommended. The patient will remain under regular clinical follow-up with endocrinology and oncology.

## Discussion

Pheochromocytoma has often been described as ‘the great mimic’ due to its presentation with myriad symptoms and diverse locations. The case highlights the diagnostic challenge posed by PPGL syndromes, as the patient presented without any symptoms of catecholamine excess and was incidentally found to have bilateral adrenal lesions. Moreover, this case emphasizes the utility of 131I-MIBG scintigraphy in the management of PPGL. The sensitivity of the 131I-MIBG scan is reported to range between 85% and 88% for pheochromocytoma [1]. In our patient, the MIBG scan showed abnormal uptake only at the site of the left-sided adrenal mass. She underwent excision of the left renal hilar paraganglioma, thus preserving bilateral adrenocortical function.

Guidelines recommend genetic testing for patients with features that indicate a high likelihood of a hereditary cause for PPGL, including positive family history, syndromic features, and multifocal, bilateral, or metastatic disease [2]. Genetic predisposition to PPGLs is well established, with up to 40% of patients with PPGLs reported to carry a germline mutation [3]. Several genes have been identified in the pathogenesis of PPGLs including RET proto-oncogene, von Hippel-Lindau disease tumor suppressor gene (VHL), neurofibromatosis type 1 tumor suppressor gene (NF1), genes encoding the succinate dehydrogenase (SDH) complex subunits [ SDHB, SDHC, SDHA and SDHD], the gene encoding the enzyme responsible for the flavination of SDHA (SDHAF2 or hSDH5), and the newly described TMEM127 and MAX tumor suppressor genes [4,5]. Germline mutations in the succinate dehydrogenase genes are the most prevalent of the genetic abnormalities which have been reported. The SDHA gene is a tumor suppressor gene and codes for one of the four subunits of the succinate dehydrogenase enzyme [6]. The genetic link between SDHA and PPGLs syndrome was established relatively recently in 2010 [7].

Compared to SDHB and SDHD, PPGL-associated SDHA mutations have been described in a relatively smaller number of patients worldwide; a nationwide study reported that pathogenic germline SDHA variants were identified in less than 1% of patients with PPGL (4). There is controversy regarding the need for surveillance screening in these patients, as studies show that SDHA mutation has a lower disease penetrance rate than those reported for other components of the succinate dehydrogenase complex [8]. The age-related penetrance has been reported to be 2% at age 50 years and 10% at age 70 years in non-index SDHA mutation carriers [9].

However, there is concern that SDHA mutated PPGLs have diverse phenotypes and may be at high risk of metastasis. A case series reported that of six index cases, two developed metastatic disease and one had local vascular invasion [10]. The incidence of metastatic disease in SDHB-related PPGL has been reported as 19%, with a high likelihood of developing extra-adrenal pheochromocytomas [11]. It has been reported that there is a prolonged time interval between diagnosis and development of metastases, indicating that SDHA mutated tumors are slow growing in character [12].

Moreover, studies have reported the occurrence of extra-adrenal tumors in 79% of SDHA mutation carriers, particularly with head and neck paraganglioma [13]. Reported associations of SDHA mutations include gastrointestinal stromal tumours (GIST), pituitary adenomas, renal carcinoma and breast carcinoma [10,14].

Given the uncertainty regarding the phenotypic behavior of this mutation, further prospective studies may be needed to detect the true incidence of metastasis in SDHA-related PPGL. Life-long annual follow-up is essential for patients with SDH gene mutations, as recommended by the Endocrine Society and the European Society of Endocrinology [2,15]. Surveillance is essential for the detection of extra-adrenal disease and recurrent tumors. The hereditary PPGL syndromes are inherited in an autosomal dominant manner. Thus genetic testing and counseling should be offered to the patient’s family, which can help diagnose tumors in an asymptomatic stage in the relatives of the gene carriers.

## Conclusions

We report a case of SDHA pathogenic variant in a middle-aged lady with an incidentally detected left renal hilar paraganglioma, who had a family history of breast cancer. The case highlights that the presentation at a later age does not rule out the hereditary nature of PPGL. The detection of susceptibility genes for hereditary PPGL syndromes has key implications for surveillance, as well as for genetic testing and counseling to the patient’s family. Further studies are needed to explore the risk of metastases and development of extra-adrenal tumors in patients with SDH mutations.
